# Synthesis of nanomedicine hydrogel microcapsules by droplet microfluidic process and their pH and temperature dependent release†

**DOI:** 10.1039/d1ra05207a

**Published:** 2021-11-23

**Authors:** Ran Liu, Qiong Wu, Xing Huang, Xiaoxiong Zhao, Xinhua Chen, Yonggang Chen, David A. Weitz, Yujun Song

**Affiliations:** Center for Modern Physics Technology, School of Mathematics and Physics, University of Science and Technology Beijing Beijing 100083 China songyj@ustb.edu.cn; Zhejiang Key Laboratory for Pulsed Power Translational Medicine Hangzhou 310000 China; Physics Department, School of Engineering and Applied Science, Harvard University Cambridge MA 02138 USA

## Abstract

Chitosan and alginate hydrogels are attractive because they are highly biocompatible and suitable for developing nanomedicine microcapsules. Here we fabricated a polydimethylsiloxane-based droplet microfluidic reactor to synthesize nanomedicine hydrogel microcapsules using Au@CoFeB–Rg3 as a nanomedicine model and a mixture of sodium alginate and PEG-*g*-chitosan crosslinked by genipin as a hydrogel model. The release kinetics of nanomedicines from the hydrogel were evaluated by simulating the pH and temperature of the digestive tract during drug transport and those of the target pathological cell microenvironment. Their pH and temperature-dependent release kinetics were studied by measuring the mass loss of small pieces of thin films formed by the nanomedicine-encapsulating hydrogels in buffers of pH 1.2, 7.4, and 5.5, which replicate the pH of the stomach, gut and blood, and cancer microenvironment, respectively, at 20 °C and 37 °C, corresponding to the storage temperature of hydrogels before use and normal body temperature. Interestingly, nanomedicine-encapsulating hydrogels can undergo rapid decomposition at pH 5.5 and are relatively stable at pH 7.4 at 37 °C, which are desirable qualities for drug delivery, controlled release, and residue elimination after achieving target effects. These results indicate that the designed nanomedicine hydrogel microcapsule system is suitable for oral administration.

## Abstract 2

A kind of pH and temperature dependent interpenetrating hydrogel was designed and synthesized *via* crosslinking of alginate and polyethylene-glycol grafting chitosan by genipin for encapsulated nanomedicine with controlled release.

## Graphical abstract

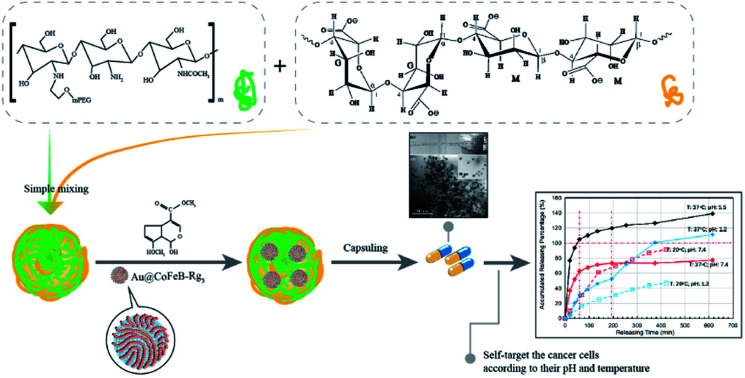

## 1. Introduction

Hydrogels are hydrophilic three-dimensional polymer networks, which can absorb and retain a large amount of water and swell without dissolving or losing their structural features.^1,2^ Hydrogels have high water permeability, they are suitable for drug delivery systems, and the embedded proteins or drugs can be released through their porous microstructures.^3–7^ Nanocomposite hydrogels are composed of polymer networks embedded with functional nanoparticles (NPs) or nanostructures.^8^ It not only improves mechanical properties, but also exhibits biological activity. In past decades, nanocomposite hydrogels have achieved unprecedented development in various fields of biomedical engineering, including drug delivery,^9–11^ wound healing,^12–15^ osteogenesis^16–19^ and bio-sensing.^20,21^

Several limitations reduce the efficacy of anticancer drugs directly administered to tumor tissues, such as short circulation time and transient high concentration due to uncontrolled drug release, unstable drug molecules and poor dispersion in the focal region, and lack of targeting.^22,23^ In 2016, Chan *et al.* analyzed the delivery efficiency of nanomedicines (NMs) to tumor sites in the last decade and found that the delivery efficiency of NMs is low, at an average of 0.7% for solid tumors, with a particularly low penetration depth.^24,25^ To achieve efficient drug delivery and high therapeutic efficiency of anticancer nanodrugs, nanodrug delivery systems of large specific surface area, appropriate pore size, and high drug-loading capacity for controlled release at the desired cell microenvironment are desirable for enhanced penetration and retention in the focal region.^26–36^ Shi *et al.*^37^ reported a nanocomposite hydrogel doped with the porous and hollow-like structure of MgSiO_3_ NPs, which can be loaded with the anticancer drug doxorubicin. The encapsulated doxorubicin can be rapidly released under the acidic tumor microenvironment.^38,39^ Therefore, exploring effective controlled-release methods and related hydrogel systems is of great significance for developing biocompatible nanomedicine encapsulation systems.

Hsing-Wen Sung *et al.* studied a hydrogel system blended with chitosan derivatives and sodium alginate using genipin as a cross-linking agent to control the release of protein drugs.^40^ Satya Prakash *et al.* studied the use of microcapsules under this system for live cell encapsulation and other delivery applications.^41^ These studies have shown that genipin cross-linked sodium alginate and chitosan can show good pH-sensitive properties, and the construction of this system is feasible. The natural polymer sodium alginate or chitosan hydrogels have received great interest owing to their biocompatibility, low toxicity, and degradability.^42–45^ These types of hydrogels undergo pH-sensitive swelling and allow diffusion of drugs through their intrinsic micropores.^46^ However, sodium alginate has poor mechanical properties and excessively high water solubility, so it must be chemically crosslinked to improve its stability in aqueous media.^47^ Chitosan can attract negatively charged proteins *via* electrostatic interactions, which results in poor drug release.^48–50^ But the low polymer concentration of the sol results in a hydrogel with poor mechanical strength. Studies have shown that polyethylene glycol-grafted chitosan (PEG-*g*-CS) can improve the mechanical and hydrophilic properties of chitosan and be conducive to drug release.^51–55^ So we use modified chitosan to construct the hydrogel system. Amino and hydroxyl groups on chitosan can form multiple bonds, but its depolymerization in acidic media hinders controlled oral administration.^56^ Genipin is used in traditional Chinese medicine for certain diseases and can spontaneously react with amino acids.^57,58^ So genipin can be used to crosslink sodium alginate and modified chitosan to prepare oral drug capsules with pH- and temperature-sensitive characteristics for controlled release.

Nanomaterials are widely used in information technology, biomedicine, catalysis and other fields. The two main types of functional nanoplatforms are plasmonic nanoparticles (mainly composed of precious metals) and magnetic nanoparticles.^59^ Nano-hybrid materials composed of precious metals and magnetic nanoparticles have broad application prospects in a wide range of fields including medical imaging, energy conversion, and disease detection due to their unique physical and chemical properties, dual-mode properties and high stability.^60^ Conjugation of nano-drugs to their surface can be used for future multi-modal diagnosis and cancer therapy. However, synthesis of nanomedicine requires very precise reaction conditions, and microfluidic process has been recognized as one of the effective methods.^61–63^ The gold nanoparticles synthesized by microfluidics can be the surface template or substrate for the deposition and growth of CoFeB shells for the construction of core–shell nanohybrids (Au@CoFeB).^64^ Boron doping metal alloy nanoparticles have the effect of clearing away heat and detoxification. Co and Fe in the Au@CoFeB are necessary dietary ingredients for humans and animals (Co can be used for VB12 synthesis; Fe can be used for heme synthesis).^65^ They can be used as supplement to nanomedicine and exhibit synergistic therapeutic effects with other metal particles. Our previous research results also show that AuCoFeB–Rg3 can be used in the treatment of cancer cells.

Au@CoFeB@Co_*x*_Fe_(1−*x*)_(OH)_2_ nanohybrids (NHs) were synthesized using a simple programmed sequencing microfluidic process, which can conjugate with the ginsenoside Rg3 to form nanomedicines, namely Au@CoFeB–Rg3.^64^ These have been used for multi-modal diagnosis and exhibit excellent cytotoxicity against various tumors. To enhance their therapeutic efficiency and administration safety, a hydrogel system for encapsulating nanomedicines into microgels should be developed to promote their oral administration and transportation to the focal area. A droplet microfluidic device was designed and fabricated for the synthesis of nanomedicine hydrogel microcapsules by using the soft LIGA process. Based on this device, nanomedicines were encapsulated into microhydrogels formed by crosslinking sodium alginate and polyethylene glycol (PEG)-*g*-chitosan with genipin. Their pH- and temperature-dependent release kinetics were studied by measuring the mass loss of small pieces of thin films formed by the nanomedicine-encapsulating hydrogel in buffers with pH 1.2, 7.4, and 5.5, which correspond to the pH levels in the stomach, gut and blood, and cancer microenvironment, respectively, at 20 °C and 37 °C, which correspond to the storage temperature of hydrogels and normal body temperature, respectively. And we also used ICP-MS spectroscopy to further study the release kinetics.

## 2. Experimental

### 2.1 Synthesis of NHs

#### Synthesis of Au@CoFeB@Co_*x*_Fe_(1−*x*)_(OH)_2_ NHs

Core–shell Au@CoFeB@Co_*x*_Fe_(1−*x*)_(OH)_2_ NHs were synthesized using our sequenced simple programmed microfluidic process through the metal salt reduction and co-precipitation reaction.^64^ Briefly, a two-step microfluidic method was used to prepare NH_S_. First, a simple programmed microfluidic method was used to prepare an ultra-small CoFeB NP solution. Then, Au@CoFeB NPs were synthesized by depositing CoFeB NPs on the surface of Au NPs through surface coating and epitaxial growth. In the first step, the metal salt solution and reducing solution were drawn into syringes (1) and (2) and pumped into the Y-type joint, respectively, which caused a rapid reducing reaction, delayed transient nucleation, and controlled growth to form ultra-small CoFeB nanoparticles, as shown in Fig. S1 of the ESI.† Microfluidic synthesis was performed at 120 °C, with a flow rate of 3 mL min^−1^, under the protection of a nitrogen atmosphere, which helps complete the reduction reaction and enables rapid nucleation. The solution was collected; the corresponding proportion of chloroauric acid was weighed and dissolved in the same volume of *N*-methyl-2-pyrrolidone (NMP) as the solution. Then, Au@CoFeB NPs were synthesized through microfluidic synthesis. Before this step, a certain amount of NaBH_4_ was dissolved in the product receiver to quench excess reducing agent. Co and Fe on the surface of NHs are easily oxidized in aqueous solution and form several hydroxyl ligands (–OH) on the surface, leading to the generation of Au@CoFeB@Co_*x*_Fe_(1−*x*)_(OH)_2_ NHs.

### 2.2 Synthesis of nanomedicines

#### Synthesis of Au@CoFeB@–Rg3 nanomedicines

Au@CoFeB–Rg3 nanomedicines were synthesized using sequential surface and ligand modification or activation and *in situ* coupling.^66,67^ Briefly, surface modification and activation of NPs was performed using the amino-silane coupling agent (3-aminopropyl)trimethoxysilane (APTMS) and the bi-functional amine-active cross-linker disuccinimidyl suberate (DSS). Then, *in situ* conjugation with the pre-modification or activation of the drug ginsenoside Rg3 was performed using APTMS. Surface hydroxyl groups on Au@CoFeB@Co_*x*_Fe_(1−*x*)_(OH)_2_ NHs were modified to amino groups by APTMS and then were activated using DSS. Surface-activated NPs were conjugated *in situ* using ginsenoside Rg3 with pre-activated hydroxyl groups by APTMS, thereby leading to the formation of Au@CoFeB–Rg3 nanomedicine.

### 2.3 Fabrication of droplet microfluidic devices

**Fig. 1:**
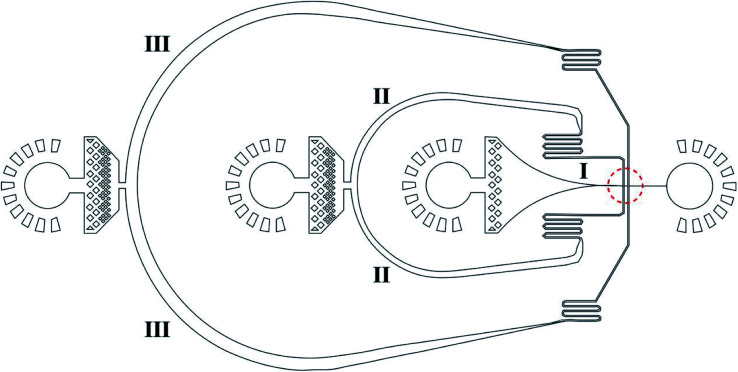
Microstructure of the droplet microfluidic device for hydrogel microdroplet generation to encapsulate nanomedicines in hydrogel microcapsules. The diameter of the arrow-shaped channel can be 8–30 μm (8 μm in this study).

Fig. 1 shows a design of the droplet microfluidic device for hydrogel microcapsule synthesis. These types of poly(dimethylsiloxane) (PDMS) devices are fabricated using traditional soft lithography based on SU-8 (a negative photoresist, MicroChem) molds.^68,69^ To fabricate the microfluidic device, the SU-8 mold was first prepared on the silicon wafer using UV photolithography.^70^ Then, the uniform pre-mixed 10 : 1 mixture of Sylgard 184 PDMS (Dow Corning, Midland, MI, USA) and cross-linker was poured onto the SU-8 mold and cured in an oven at 65 °C for more than 2 h after vacuum degassing. PDMS microdevices were extracted with a metal scalpel and punched with a 0.75 mm or 1.0 mm biopsy punch (Harris Uni-Core, Ted Pella, Inc., Redding, CA) to create inlets and outlets. PDMS microdevices were then bonded to a glass slide after an oxygen-plasma activation of both surfaces and baked at 65 °C for 1 h. Finally, the device was functionalized through hydrophobic treatment using Aquapel (PPG Industries, Pittsburgh, PA, USA) to render the channel surface hydrophobic.

### 2.4 Encapsulation of nanomedicines

#### Encapsulation of Au@CoFeB–Rg3 nanomedicines

The nanomedicine–polymer solution for nanomedicine hydrogel microcapsules was prepared by first dissolving 20 mg Au@CoFeB–Rg3 in 4 mL phosphate-buffered saline (PBS). Then, 20 mg sodium alginate (Sigma-Aldrich, 250 cps for a 2% aqueous solution at 25 °C) and 20 mg PEG-*g*-chitosan (Sigma-Aldrich, ≥60% [titration] crystalline, degree of polymerization ≥400) were added to 4 mL PBS containing Au@CoFeB–Rg3 and mixed until a uniform solution of 1.0 wt% polymer and 0.5 wt% nanomedicine was formed. Then, 0.68 mg of the cross-linker “Genipin” (0.75 mM) was added to the nanomedicine–polymer mixture, mixed uniformly, and stored at 4 °C.

Au@CoFeB–Rg3 nanomedicine microcapsules were synthesized using the droplet microfluidic device to form hydrogel droplets. The procedure to generate hydrogel droplets in an oil emulsion uses the devices described. The outer phase (channel III in Fig. 1) is HFE-7500 (3M, MN, USA) with 1.0% surfactant (perfluorinated polyethers–polyethyleneglycol, RainDance Technologies, MA, USA) oil. The inner phase (channel I in Fig. 1) comprises the nanomedicine–polymer–genipin solution for the formation of hydrogel droplets. The flow of the inner phase is sheared by the outer oil phase at the cross-junction (red dotted circle), forming monodisperse droplets of hydrogel drops in the end part of channel I connecting to the orifice. The hydrogel drops are collected in a vial and crosslinked to the nanomedicine hydrogel at room temperature for more than 2 days. To confirm crosslinking, drops of PFO (1H, 1H, 2H, 2H, perfluoro-octanol (CF_3_(CF_2_)_5_(CH_2_)_2_OH)) were added to droplets after crosslinking. If no significant loss of hydrogel droplets (merging of hydrogel microcapsules) was observed, crosslinking the hydrogel as microcapsules for oral administration was considered sufficient. Thereafter, more PFO and some water were added to the sample vial. The mixture was vortexed and centrifuged at 800 × *g* for 1–2 min. The PFE oil at the bottom was carefully removed using a pipette. More water was added to the sample; the sample was vortexed and centrifuged to obtain hydrogel drops. The PFE oil at the bottom was removed using a pipette. The washing step was repeated three times to ensure removal of PFE oil and obtain purified hydrogel drops. A drop of the nanomedicine hydrogel was placed on a glass slide using a pipette and evaluated through optical microcopy to check the stability and dispersion of nanomedicine hydrogel microcapsules during drying.

### 2.5 Characterization of the pH-dependent release kinetics of encapsulated nanomedicines from hydrogels

To study the controlled release of encapsulated nanomedicines from the nanomedicine hydrogel, we used the weighting method to investigate the rates of release of nanomedicine from the dry thin films formed by nanomedicine hydrogels. This is because synthesis of an adequate amount of the nanomedicine hydrogel sample using current single-line droplet microfluidics was difficult for a precise study. The nanomedicine hydrogel solution was cast into a Petri dish and dried at 60 °C for more than 2 days in an air-circulating oven to form 10–20 μm thin films, which were then cut into 3–10 mm × 3–10 mm pieces for future use. The dry thin films can be used to study the re-swelling characteristics of the synthesized microcapsules by simulating the digestive process after oral administration. In addition, the cost-effectiveness and long-term storage of nanodrugs was improved after these capsules were dried, which was similar to dry food.

Furthermore, 30–50 mg of dry nanomedicine hydrogel thin films were added to PBS of a certain pH, namely 1.2, 5.5, and 7.4, similar to the pH values of the stomach, microenvironment of cancer cells, and microenvironment of healthy cells in the gut and blood, respectively, at a certain temperature (here, 20 °C for storage and 37 °C for the body temperature of healthy individuals) in a small thermostat; then, placed in a shaker for 5 min, 10 min, 20 min, 30 min, 40 min, 60 min, 90 min, 120 min, 150 min, 180–240 min, 280–360 min, 420 min, or 620 min. Then the buffer was removed completely with a pipette, the nanomedicine hydrogel pieces were washed rapidly three times using nanopure water under shaking at room temperature, dried at 60 °C in an air-circulating oven for 2 h until constant weight (almost the same weight at least three times with error less than ±5% for a 10 min drying interval). Then, their release kinetics were studied by plotting release time-dependent accumulated mass loss at a certain temperature and pH.

We also used ICP-MS spectroscopy (Agilent 7800, Agilent) to further study the release kinetics. ICP spectroscopy can perform qualitative, semi-quantitative and quantitative analysis of a variety of elements in materials. Its qualitative analysis is usually accurate and reliable, and it is the only method in atomic spectroscopy that can be used for qualitative analysis. We placed the nano-medicine hydrogel dry films in solutions with pH 1.2, 5.5, 7.4, and soaked at 37 °C for 10 min, 20 min, 40 min, 60 min, 120 min, 180 min, 300 min. After soaking for a period of time, the supernatant was sucked away, and finally the metal element content released in the supernatant was directly measured by ICP-MS spectroscopy to calculate the release efficiency.

### 2.6 Morphology and swelling kinetics characterization of nanomedicine encapsulating hydrogel thin films

After the nanomedicine encapsulating hydrogel thin films were swelled in deionized water reaching a balance, they were dried by a freeze dryer and then they are broken across the cross-section. One piece of the thin film and the cross-section sample were faced up and fixed on the sample holder, and then spray-covered with a layer of gold for microstructure observation *via* a scanning electron microscope (Supra 55, ZEISS). The swelling kinetics of the nano-medicine hydrogel thin films were tested by the gravimetric method. The dried hydrogel with a mass of *m*_1_ was immersed in a PBS buffer with a pH of 7.4. The hydrogel thin films were taken out at 10, 20, 40, 60, 120, 180, 240 and 300 min. Their surface residue water was absorbed with a piece of filter paper and their masses (*m*_2_) were weighed by a balance (ME204, 0.1 mg, METLER TOLEDO). The formula for calculating the swelling rate is SR = (*m*_2_ − *m*_1_)/*m*_1_.

## 3. Results and discussion

**Fig. 2:**
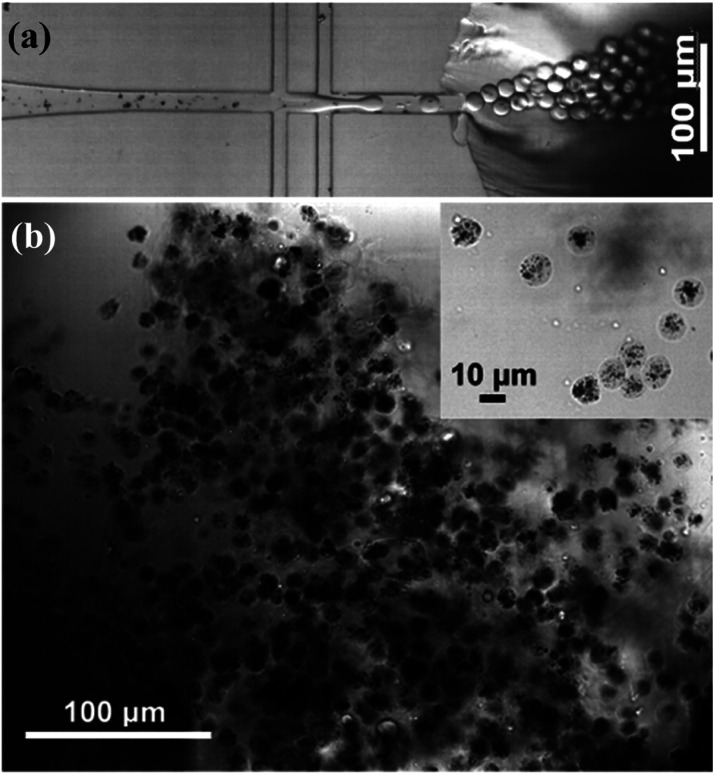
(a) Multi-junction droplet microfluidic reactor for nanomedicine microcapsule synthesis. (b) Bright-field optical image of synthesized nanomedicine microcapsules.

Fig. 2a shows the formation of Au@CoFeB–Rg3 nanomedicine microhydrogels using droplet microfluidics, observed using a white light optical microscope. Uniform hydrogel microdrops are formed at the channel junction between the continuous outer HFE-7500 oil phase (channel I in Fig. 1) and nanomedicine–polymer–genipin aqueous buffer phase (channel II in Fig. 1) subjected to be sheared to droplets by the outer oil phase. These microcapsules had good stability and dispersion in the oil phase. Fig. 2b is the optical image of synthesized nanomedicine microcapsules that were transferred to the aqueous phase after crosslinking for more than 2 days. They were also stable and well-dispersed in the aqueous solution, showing a uniform diameter of ∼10 μm. The nanomedicine was successfully encapsulated into each hydrogel microcapsule (dark dots in each microcapsule: top right magnified image), although they formed aggregates during the formation of hydrogel microcapsules and the crosslinking effect owing to enhanced local concentration and magnetic dipole effects. The average diameter of each nanomedicine is 6.6 ± 0.7 nm, which can be enriched in tumor tissue through the EPR effect. Nanoparticles can also autonomously target tumor tissues by using their special microenvironment.

**Fig. 3:**
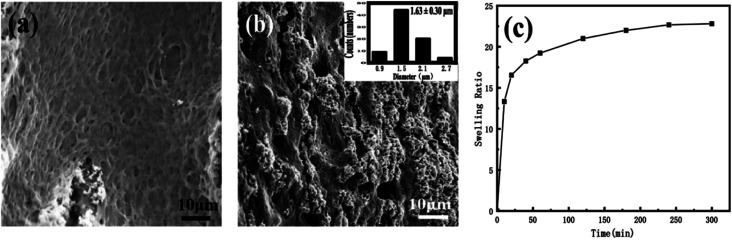
(a and b) SEM images of different cross-sections of nano-medicine hydrogel (the inserted is the histogram of the size distribution). (c) Swelling properties of nano-medicine hydrogels.

After the nanomedicine hydrogel was swelled and balanced in deionized water, the image was observed with SEM. As shown in Fig. 3a and b, the hydrogel thin films preserve microporous interpenetrating network, formed by the cross-linking of PEG-*g*-chitosan and alginate *via* genipin. The pore size is statistically analyzed by more than 200 random selected pores in the cross-section of the thin films (Fig. 3b), giving a mean diameter of 1.63 μm ± 0.30 μm (inserted image in Fig. 3b). Fig. 3c is the swelling rate of the nanomedicine hydrogel thin films. The swelling rate depends on the degree of cross-linking between gel materials. The swelling rate of the resulting hydrogel thin films will be reduced significantly with the increase of the cross-linking degree of the nanomedicine hydrogel. When the pH is 7.4, the swelling rate of the nanomedicine hydrogel is 23%, and the loading rate is 33% based on the total dry mass of the nanomedicine hydrogel.

This result indicates successful formation of well-dispersed water stable nanomedicine microcapsules through crosslinking of a mixture of sodium alginate (Na(C_6_H_7_O_6_)_*n*_, see molecule structure in Fig. S2a in the ESI†) and PEG-*g*-chitosan (see molecule structure in Fig. S2b†) by genipin (C_11_H_14_O_5_, *M*_w_ = 226.2259, see molecule structure in Fig. S2c†). Genipin is derived from the fruit of *Gardenia jasminoides* Ellis, which has sufficient active hydroxyl and carboxyl groups, which can be used as a natural biocompatible crosslinking reagent, with a coupling reaction similar to glutaraldehyde.^71–75^ Particularly, it can be used to crosslink polymers with amine groups, such as chitosan (Fig. S2d†), and form strong interactions with polymers rich in hydroxyl and carboxyl groups, such as alginate or PEG, through a combination of hydrogen bonds, ionic bonds, and van der Waals forces (Fig. S2e†).

**Fig. 4:**
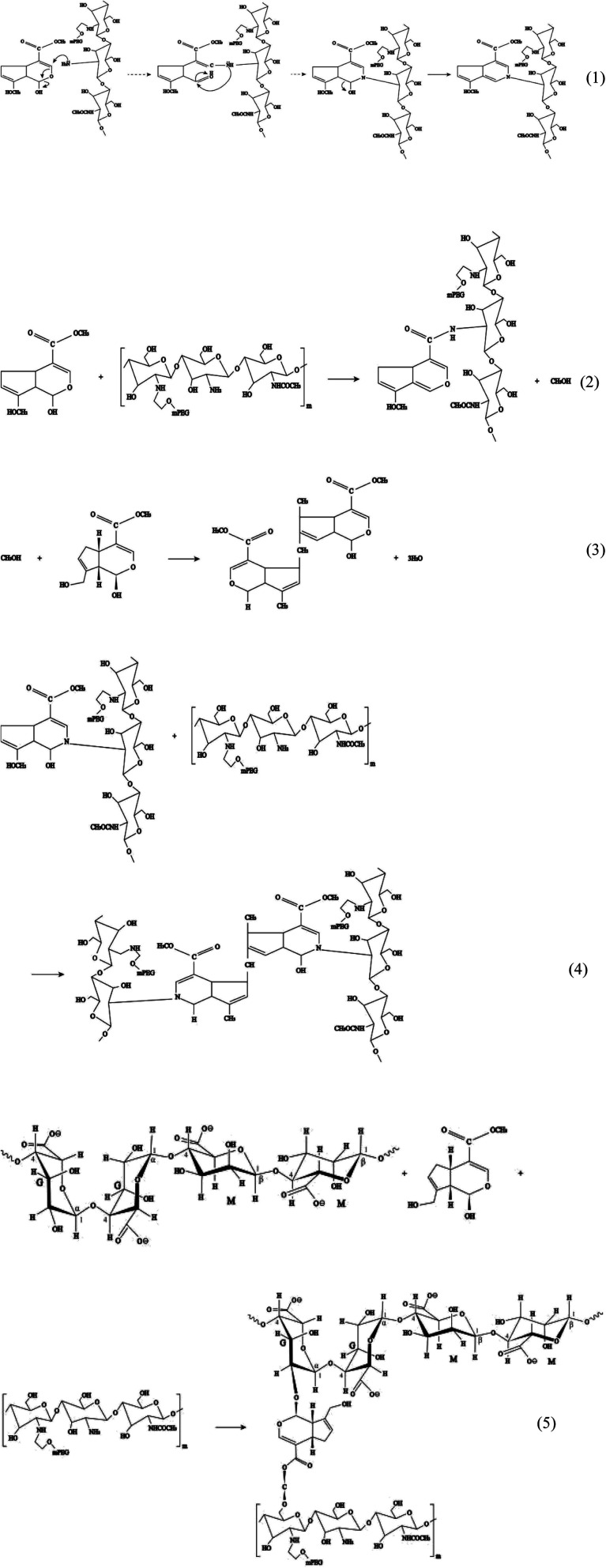
Polyethylene glycol (PEG)-*g*-chitosan coupling with genipin.

The hydrogel system in this study was constructed using crosslinking sodium alginate and PEG-*g*-chitosan. PEG-*g*-chitosan can be crosslinked by genipin through two types of coupling reactions. The first step is the ring opening reaction that occurs through the nucleophilic attack of active olefinic carbon in genipin by amino groups in PEG-*g*-chitosan (reaction (1) in Fig. 4). The second involves an S_N_2 nucleophilic substitution reaction, or the formation of amide bonds between active ester side groups and amine groups in PEG-*g*-chitosan (reaction (2)).^76,77^ During the coupling reaction, genipin can self-couple to form dimers and further self-polymerize (reaction (3)), which provides the chitosan framework with high strength and intrinsic fluorescence. The high amount of methanol produced in reaction (2) promotes the self-polymerization of genipin, leading to the formation of heterocyclic aromatic amines (reaction (4)) among PEG-*g*-chitosan.^78,79^ Particularly, it is suitable for encapsulating medicines and biological agents, which cannot be treated at a high temperature, as well as organic solvents, because all crosslinking reactions can be completed under ambient temperatures in aqueous solution.

In addition, genipin can react with hydroxyl groups in sodium alginate and PEG-*g*-chitosan/alginate (reaction (5)), further increasing the degree of crosslinking. The resulted interpenetrating microporous network can enhance the chemical stability, mechanical strength, and biocompatibility of hydrogel microcapsules, thereby favoring to the drug-loading capacity and transportation safety.

Oral administration is a common and convenient route for drug administration. Oral controlled-release preparations are often restricted by the gastrointestinal environment. During transportation, drugs enter the weakly alkaline intestine from the strongly acidic stomach.^80^ Only by ensuring that the drugs are not released or scarcely released under the strongly acidic conditions of the stomach, can drugs be absorbed by intestinal capillaries and transported through the blood to the liver or other lesions. During transport in the blood (pH = 7.4, *T* = 37 °C), drugs should be released as slowly as possible to reduce drug wastage and circumvent destruction by macrophages. Then, they undergo accelerated release after reaching the lesion. Therefore, we simulated the temperature and pH environment of the stomach, intestine, blood, and tumor site during oral administration. Simultaneously, we studied whether the medicine could be accumulated in the patient for a period after administration of the medicine with room-temperature water. We also investigated the slow-release effect in an environment that simulated stomach and blood pH at room temperature. Our simulated temperature values were 37 °C (body temperature) and 20 °C (room temperature), and simulated pH values were 7.4 (intestine and blood), 5.5 (tumor site), and 1.2 (stomach).

**Fig. 5:**
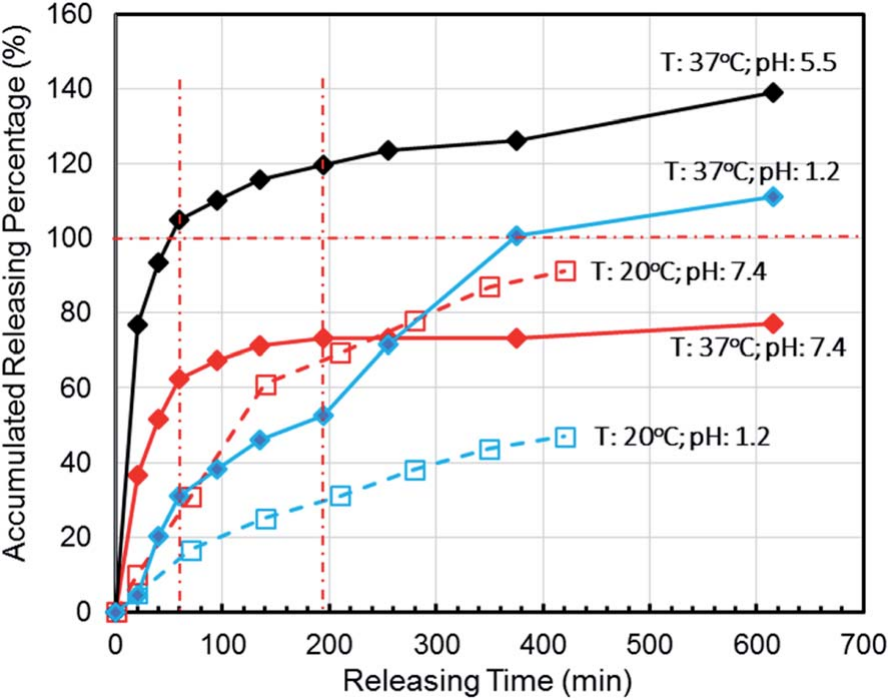
Effects of controlled nanomedicine release from hydrogel thin films in PBS at different pHs and temperatures by simulating the pHs and temperatures of the stomach (pH = 1.2 at 37 °C), small intestine (pH = 7.4 at 37 °C), and cancer cell microenvironment (pH = 5.5 at 37 °C) in correlation with the release at an ambient storage temperature (20 °C) under different pH (1.2 and 7.4) conditions for oral administration.

**Fig. 6:**
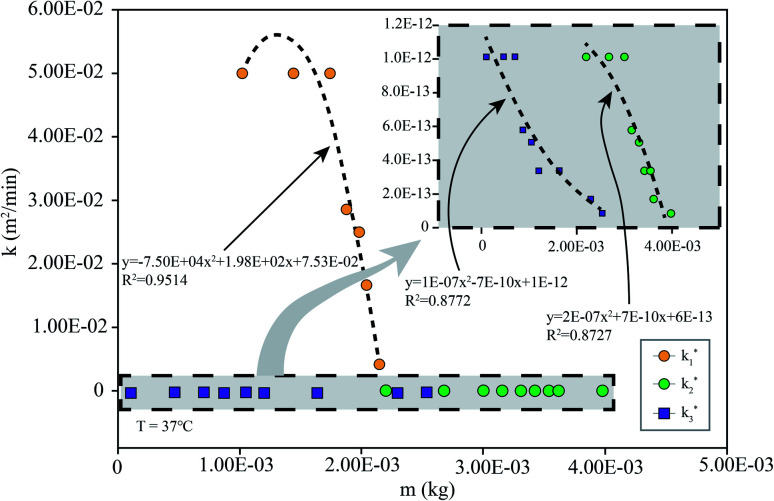
*k* simulated by the model (eqn (6)) at different *m*: the simulation is the diffusion coefficient at different pHs at 37 °C (the corresponding pH of *k*_1_ is 7.4, that of *k*_2_ is 5.5, and that of *k*_3_ is 1.2).

To simulate the presence of the drug in the stomach, the drug was released at a controlled pH of 1.2 at 37 °C. As seen in Fig. 5, the release efficiency of the nanodrug only reached 15% in 30 min. Fig. 6 shows the changing trend of the diffusion coefficient with the change of mass at 37 °C. The rate of change of mass with time is equal to the negative value of the change of diffusion flux with distance, which is consistent with Fick's second law, as shown in eqn (6):6
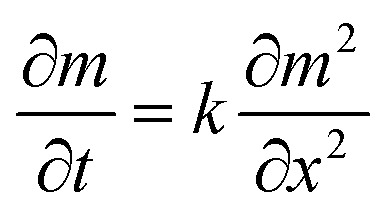
where *m* is the quantity of the product (kg), and *x* is the distance to which the drug is released (m).

To simulate the presence of the drug in the blood and tissues, the drug was released at a controlled pH of 7.4 at 37 °C. The release efficiency was as high as 55% in 30 min. The entire process conformed to Fick's second law. Simultaneously, when the drug reached the tumor site, the pH value was 5.5. The release efficiency was as high as 95%, which favors the preparation of orally administered nanomedicine microcapsules. The residence time of the drug in the stomach was approximately 30 min. Comparing the release rate of the previous 30 min revealed that a very small amount of the drug was released into the stomach. Therefore, the drug can pass through the small intestine, reach the tumor site, and be released in large quantities. Interestingly, the release percentage at 37 °C was higher than 100% after 1 h when the pH was 5.5 because hydrogels start decomposing at this pH, thereby leading to the dissociation of crosslinked networks into soluble species (non-crosslinked alginate and/or PEG-*g*-chitosan molecules) as the drug is released. The free alginate and/or PEG-*g*-chitosan chains redissolve into the aqueous buffer solution, thereby increasing the amount of overall released materials to more than the total nanomedicine mixed into the hydrogels. This results in an accumulated release percentage of more than 100%. The drug gradually turned gray, and eventually the buffer solution flocculated. This phenomenon was desirable, because rapid degradation of the hydrogels accelerates the release of nanomedicines into the tumor microenvironment and increases their concentration around the tumor cells as well as their general utilization efficiency.

**Fig. 7:**
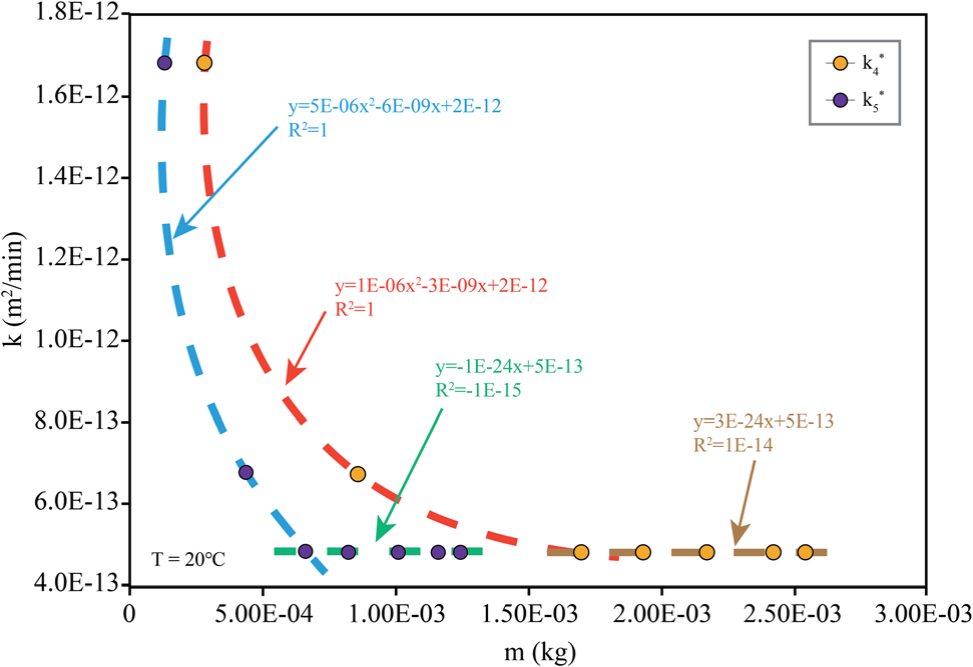
*k* simulated by the model (eqn (6)) at different *m*: simulation is the diffusion coefficient at different pHs at 20 °C (the corresponding pH of *k*_4_ is 7.4 and that of *k*_5_ is 1.2).

When the drug reaches the pH environment of the stomach and small intestine at a simulated room temperature of 20 °C, the entire release process matches Fick's second law as shown in Fig. 7. After the drug released, the drug concentration gradually decreased. And under the influence of the non-physical effect of the hydrogel and the drug, the later diffusion tended to be stable. So the nanomedicine can only be released into the solution when the hydrogel is degraded. In the first 200 min, the release rate is decreased at the reduced temperature (20 °C), which is less than 10% at pH 1.2 and less than 15% at pH 7.4. Compared to 37 °C, the release efficiency is much lower. This is based on the relationship between diffusion coefficient and temperature as eqn (7):

7 *k* = *A* exp(*E*_a_/*RT*)

According to eqn (7), the activation energy required for nanomedicine is estimated to be 2.26 × 10^4^ J mol^−1^ based on the current data, which signifies that the more the activation energy required, the slower the drug release. From this result, it can be deduced that the drug can be stored in ambient conditions with little loss of the crosslinking even in high-humidity conditions. Therefore, by using genipin as a crosslinking agent, nanomedicines can be safely encapsulated in the crosslinked alginate/PEG-*g*-chitosan to form relatively stable nanomedicine microcapsules, which are desirable for oral administration.

**Fig. 8:**
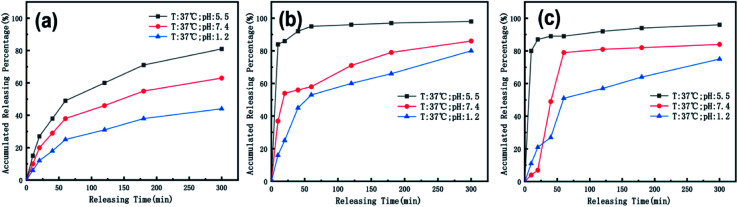
The temperature is 37 °C, and the release efficiency of (a) Au, (b) Co, (c) Fe in the nanomedicine hydrogel are at pH 1.2, 5.5, and 7.4.

We also used ICP spectroscopy to directly measure the metal release efficiency of the nanomedicine hydrogel. As shown in Fig. 8, the release efficiency trend of the metal in the hydrogel under different pH conditions is similar to the previous test results. Our nanoparticles have a core–shell structure, and Au components are wrapped inside, so compared to CoFeB alloy components, the release efficiency of Au is lower at the early 50 min. The general trend of metal particles is that the release efficiency is the highest when the pH is 5.5, and the release efficiency is the lowest when the pH is 1.2. This proves once again that our nano-medicine hydrogel microcapsules can successfully pass through the stomach, pass through the intestine, and finally reach the tumor site for mass release.

One key reason for genipin used here is that genipin is a kind of natural crosslinking agents of highly biocompatibility and varieties of biofunctions, such as inhibition of uncoupling protein 2 (UCP2), modulating proteins, antitumors, anti-inflammatory, immunosuppressive effects, antithrombotic effects and protection of hippocampal neurons.^71–73^ Cytochrome c content increases significantly in the cytosol of genipin-treated FaO cells.^74^ Activation of caspase-3 and caspase-7 leads to genipin-induced apoptosis in hepatoma cells. ROS level notably increased in Hep3B cells treated with 200 μM genipin. Genipin is both anti-tumorigenic and anti-thrombogenic.^75^ Particularly, it is of great value for reducing infection and thrombosis caused by cell necrosis. Therefore, genipin can not only be used as a cross-linking agent for nano-medicine hydrogels, but also is expected to increase the biological features of the synthesized nano-medicine microcapsules.

## 4. Conclusions

In summary, a type of nanomedicine-encapsulating hydrogel for preparing nanodrug microcapsules was designed using Au@CoFeB–Rg3 as a type of nanomedicine model and a mixture of sodium alginate and PEG-*g*-chitosan crosslinked by genipin as a type of hydrogel model that combines the advantages of both chitosan and alginate. This type of nanomedicine-encapsulating hydrogel exhibits desirable pH- and temperature-dependent releasing kinetics that are particularly suitable for oral administration: with the lowest release rate at pH 1.2 (the normal pH in stomach) and the highest release rate and rapid decomposition rate at pH 5.5 (the normal pH in the microenvironment of solid tumors) at 37 °C. This hydrogel also exhibits a relatively lower releasing rate and a stronger capacity to maintain the stable microstructures at pH 7.4 (the transportation microenvironment, such that as in the blood) than at pH 5.5 at 37 °C. Furthermore, a droplet PDMS microfluidic device was successfully developed using traditional soft lithography based on SU-8, which exhibits a remarkable ability in preparing uniform nanomedicine hydrogel microcapsules with controlled sizes for orally administrated nanomedicines with high efficiency. These nanomedicine hydrogels and synthesized microcapsules can be orally administrated effectively for safe passage through the acidic stomach, following which they will be absorbed into the blood stream through the gut system and be circulated into the tumor microenvironment, where they will release the encapsulated nanomedicines and self-decompose rapidly, fulfilling their therapeutic effects. These characteristics of the drug indicate that our drug is an autonomously targeted drug, which autonomously targets the tumor microenvironment through pH and temperature. At the same time, our drug is a polymer interpenetrating network system of highly biocompatibility, which lays on the foundation for the future as a perspective carrier of cancer drugs.

## Conflicts of interest

There are no conflicts to declare.

## Supplementary Material

RA-011-D1RA05207A-s001
