# Supplementary material for: Synthesis of nanomedicine hydrogel microcapsules by droplet microfluidic process and their pH and temperature dependent release
Source: RSC Adv. 2021 Nov 23;11(60):37814–23. doi: 10.1039/d1ra05207a (PMC9043787; doi:10.1039/d1ra05207a)
Supplement: RA-011-D1RA05207A-s001 [file RA-011-D1RA05207A-s001.pdf]

Electronic Supplementary Material (ESI) for RSC Advances.
This journal is © The Royal Society of Chemistry 2021

Synthesis of Nanomedicine Hydrogel Microcapsules through Droplet Microfluidics and their pH- and Temperature-Dependent Release Kinetics

Ran Liu[1,2‡], Qiong Wu[1,2‡], Xing Huang[3], Xiaoxiong Zhao[1,2], Xinhua Chen[2], Yonggang Chen[2], David A.Weitz[3], Yujun Song[1,2,3]*

[1] Center for Modern Physics Technology, School of Mathematics and Physics, University of Science and Technology Beijing, Beijing 100083, China; [2] Zhejiang Key Laboratory for Pulsed Power Tanslational Medicine, Hangzhou, 310000, China; [3] Physics Department, School of Engineering and Applied Science, Harvard University, Cambridge, MA 02138, USA

‡ Contributed to this article equally.

* Corresponding Author: songyj@ustb.edu.cn

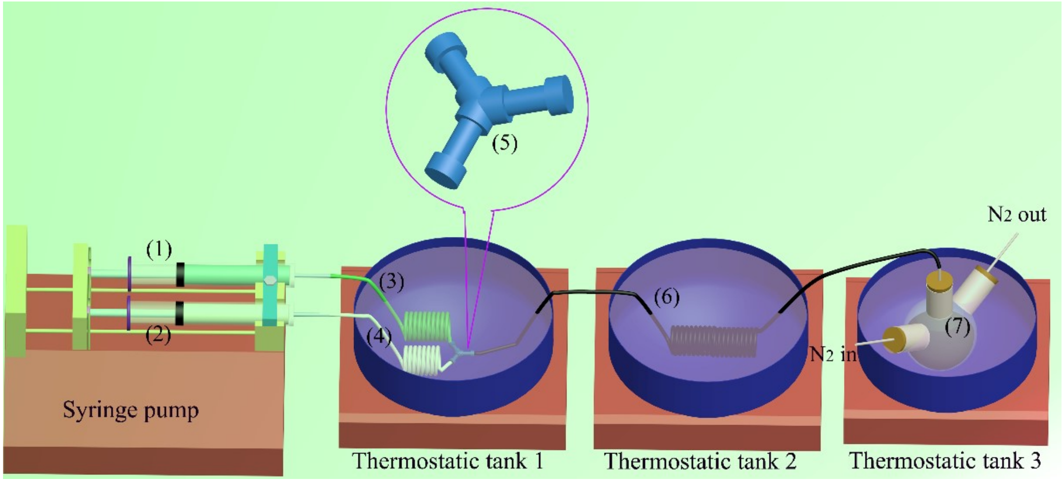

Scheme S1. Temperature programmed microfluidic process for the synthesis of NPs.

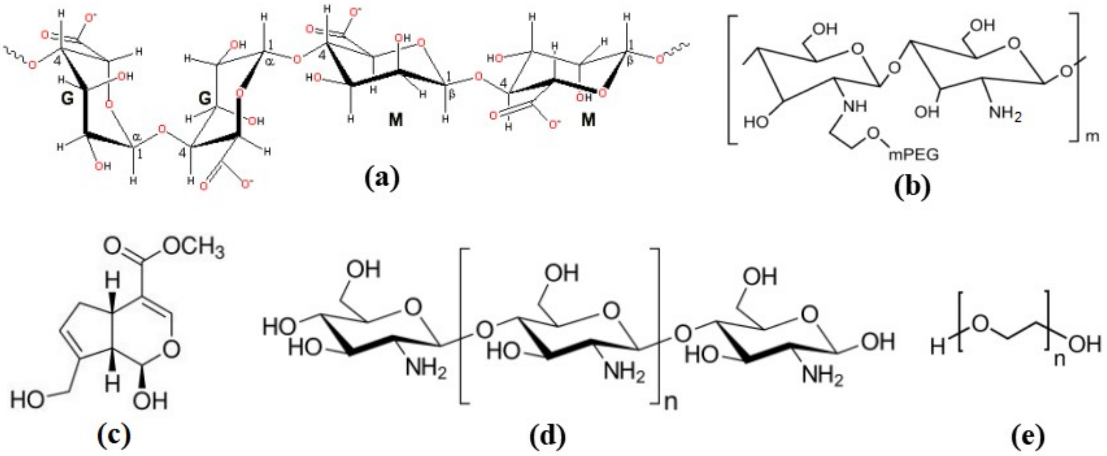

Scheme S2 Molecular structure of (a) alginate, (b) PEG-g-chitosan, (c) genipin, (d) chitosan, (e) polyethylene glycol (PEG), which have strong interactions with polymers rich in hydroxyl groups and/or amine or carboxyl groups.
